# Biomarker Testing Trends in Patients With Metastatic Colorectal Cancer Who Live in Rural Areas and Urban Clusters in the US

**DOI:** 10.1093/oncolo/oyad244

**Published:** 2023-09-08

**Authors:** Mark A Lewis, Lindsay Stansfield, John M Kelton, Christopher H Lieu

**Affiliations:** Gastrointestinal Oncology, Intermountain Medical Center, Murray, UT, USA; Pfizer, New York, NY, USA; Pfizer, New York, NY, USA; Division of Medical Oncology, University of Colorado Cancer Center, Aurora, CO, USA

**Keywords:** biomarker testing, colorectal cancer, rural area, urban clusters

## Abstract

**Background:**

There is a paucity of data on biomarker testing rates in rural populations with metastatic colorectal cancer (mCRC). To assess biomarker testing practices, oncologists in rural areas and urban clusters in the US were surveyed.

**Materials and Methods:**

A web-based survey was administered to oncologists spending ≥40% of their time practicing in rural areas or urban clusters and who had treated ≥2 patients with stage IV mCRC in the prior month.

**Results:**

Ninety-nine oncologists completed the quantitative survey and 17 the qualitative interview. Among respondents, 97% reported ordering biomarker tests. Oncologists reported testing for *KRAS*, *NRAS*, *BRAF*, *HER2*, and mismatch repair deficiency/microsatellite instability in 72%, 65%, 63%, 56%, and 66% of patients with metastatic disease, respectively. Forty-one percent reported performing reflex testing. The most cited testing barriers were lack of insurance coverage, insufficient tissue samples, and long turnaround times.

**Conclusion:**

Further assessment of rural testing practices is needed.

## Teaser

In this study, oncologists in the US were surveyed to investigate practice patterns related to biomarker testing in rural areas and urban clusters, uncover obstacles to biomarker testing, and understand the role of telehealth in metastatic colorectal cancer.

## Introduction

With approximately 153 000 new diagnoses and 53 000 deaths estimated in 2023, colorectal cancer (CRC) is the fourth most common cancer and the second most common cause of death from cancer in the US.^1^ The overall death rate for CRC decreased 19% between 2007 and 2017.^2^ Between 1995 and 2013, the incidence of CRC decreased 32.2% among urban populations but only 21.8% among rural populations.^3^ From 2007 to 2017, patients living in rural areas experienced smaller decreases in rates of death from all types of cancer than those living in urban areas (4% vs 22%).^2^

The National Comprehensive Cancer Network 2023 clinical practice guidelines for metastatic colon and rectal cancer recommend testing for *BRAF* and *RAS* mutations, mismatch repair deficiency/microsatellite instability (MMR/MSI), and *HER2* amplification in patients with metastatic CRC (mCRC) to aid treatment decisions, either individually or as part of a next-generation sequencing panel. *NTRK* fusions are extremely rare and may be more frequently found in patients with MMR deficiency.^4,5^ Despite these recommendations, testing rates fall short. In addition, there is a paucity of data on biomarker testing rates in rural populations with mCRC.

We surveyed oncologists in the US to investigate practice patterns related to biomarker testing in rural areas and urban clusters, uncover obstacles to biomarker testing, and understand the role of telehealth in mCRC.

## Results

### Demographics of Oncologists

**Figure 1.**
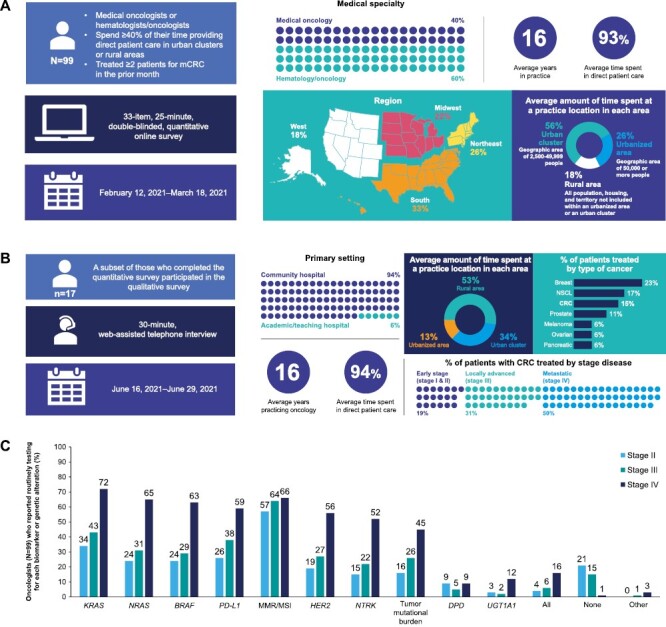
Demographics of oncologists surveyed in the (**A**) quantitative survey and (**B**) qualitative interviews. (**C**) Self-reported biomarker and genomic alterations testing practices of oncologists. Three oncologists did not sign the informed consent form. (**A**) Adapted from a poster presented at the American Society of Clinical Oncology (ASCO) Quality Care Symposium; September 24-25, 2021; Boston, MA, USA, and virtual; © the authors. (**B**) Adapted from poster 70 presented at the American Society of Clinical Oncology Annual Gastrointestinal Cancers Symposium (ASCO-GI); January 20-22, 2022; San Francisco, CA, USA, and virtual; © the authors. Abbreviations: BRAF, v-raf murine sarcoma viral oncogene homolog B1; DPD, dihydropyrimidine dehydrogenase; HER2, human epidermal growth factor receptor 2; KRAS, Kirsten rat sarcoma virus; MMR/MSI, mismatch repair deficiency/microsatellite instability; NRAS, neuroblastoma ras viral oncogene homolog; NTRK, neurotrophic tyrosine receptor kinase; PD-L1, programmed cell death ligand 1; UGTA1, uridine diphosphate glucuronosyltransferase 1A11.

**Figure 2.**
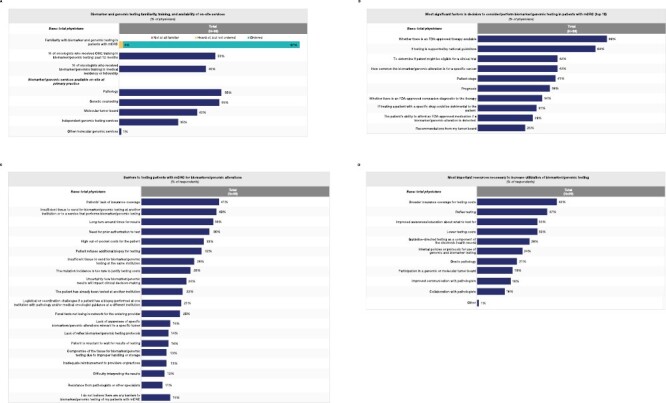
(**A**) Biomarker and genomic testing familiarity, training, and availability of on-site services. (**B**) The 10 most significant factors in the decision to consider or perform biomarker/genomics testing in patients with mCRC. (**C**) Barriers to testing patients with mCRC for biomarkers/genomic alterations. (**D**) The most important resources necessary to increase biomarker/genomic testing. CME, continuing medical education; FDA, US Food and Drug Administration; mCRC, metastatic colorectal cancer. (**C**) and (**D**) Adapted from a poster presented at the American Society of Clinical Oncology (ASCO) Quality Care Symposium; September 24-25, 2021; Boston, MA, USA, and virtual; © the authors.

During the observation period, 99 oncologists completed the quantitative survey (Fig. 1A). Of these, 17 completed the qualitative survey (Fig. 1B). The oncologists who responded spent most of their time practicing in urban clusters rather than urbanized areas and rural areas. In terms of geographic distribution, most were based in the South, followed by the Northeast, Midwest, and West regions (Fig. 1A). Almost all of the oncologists reported that they had ordered biomarker tests for their patients with mCRC; around one-third reported using independent genomic testing services (Fig. 2A).

### Practice Demographics

The oncologists reported that most of the patients in their practice were White and lived an average of 19 miles from the location for regular check-ups (Supplementary Fig. 1). Medicare and private/commercial insurance were the most common types of insurance in the practices surveyed.

### Biomarker Testing (Self-Reported Testing Practices)

Oncologists reported testing biomarkers across disease stages II to IV and for specific biomarkers (eg, *KRAS*, *NRAS*, *BRAF*, *HER2*, PD-L1, MMR/MSI) (Fig. 1C). Among respondents, 41% said their primary practice had reflex biomarker/genomic testing in place. Of those with reflex testing, the biomarkers most often tested reflexively were PD-L1 (62%), *KRAS* (60%), MMR/MSI (52%), and *BRAF* (46%). DNA-based next-generation sequencing testing was the most commonly reported biomarker/genomic alteration testing method used for patients with mCRC (44%-63%), except for PD-L1, which was assessed by immunohistochemistry (45%).

The most significant factors in deciding whether to perform biomarker testing in patients with mCRC included the availability of US Food and Drug Administration–approved therapy (69%), testing recommendations from national guidelines (63%), eligibility for a clinical trial (42%), commonality of a biomarker/genomic alteration for a specific cancer (42%), stage of cancer (41%), prognosis (38%), and the ability of the patient to afford the testing (29%) (Fig. 2B). The majority of oncologists (87%) agreed or strongly agreed that the value of biomarker testing increases if there is an US Food and Drug Administration-approved therapy for a specific biomarker/genomic alteration.

Interestingly, the majority of oncologists (80%) thought biomarker testing rates were optimal, reporting that laboratories for testing are easily accessible, reputable, and have an established, streamlined ordering process.

### Barriers to Biomarker Testing

The most commonly reported barriers to testing were lack of insurance coverage (41%), insufficient tissue samples to send to another institution/service that performs biomarker/genomic testing (40%), long turnaround times for results (38%), and high out-of-pocket costs (33%) (Fig. 2C).

Oncologists stated that the most important resources required to increase utilization of biomarker/genomic testing were broader insurance coverage for testing costs (42%), use of reflex testing (37%), improved education/awareness of what to test for (32%), and lower testing costs (32%) (Fig. 2D). Improved communication (18%) and collaboration with pathologists (16%) were also mentioned.

### Use of Telehealth

Oncologists reported that the percentage of oncology patients who were seen via telehealth/virtual visits nearly quadrupled (from 9% to 34%) during the COVID-19 pandemic, with most visits using video (72%) and/or phone (64%). Although 56% of the oncologists agreed that telehealth can improve biomarker and genomic rural testing rates, 81% of these noted barriers, including patients’ lack of technology equipment (56%), patients being disengaged or unwilling to use telehealth (37%), and issues with Wi-Fi connections (23%). Interestingly, nearly 20% of oncologists did not perceive any barriers to implementing telehealth at oncology practices.

## Discussion

To our knowledge, this survey is the first to explore mCRC biomarker testing specifically in rural and urban cluster settings. Consistent with the literature, our survey demonstrated that biomarker testing rates for mCRC are suboptimal and do not conform to national guidelines.^4,5^

In rural communities, barriers may be more pronounced than in urban communities due to geographic or financial reasons, limited access to comprehensive cancer care, and other reasons, regardless of cancer type or stage.^6^ Barriers to testing may arise from insufficient tissue samples, financial hardships related to testing costs, clinicians’ lack of knowledge or awareness of the need to test, communication barriers among the oncology care team, misinterpretation of the value and importance of testing, lack of standardization in biomarker testing, lengthy and complex reports, low reimbursement, lack of education on guidelines, and long turnaround times for results.^7,8^ Our survey responses agreed with some of these previously published points. Oncologists cited lack of patient insurance coverage, insufficient tissue samples, long turnaround times for results, and high out-of-pocket costs as playing a role in the barriers to biomarker/genomic testing in patients from rural areas and urban clusters.

Telehealth creates a wider catchment area for patients to access health and medical services, accentuating the need to overcome the logistical hurdles. During the survey, which occurred during the COVID-19 pandemic at varying levels of state lockdowns, the relaxation of longstanding regulatory and reimbursement restrictions on telehealth helped provide leeway for providers to give virtual healthcare to patients (eg, under Medicare).^9^ For example, telehealth for genetic counseling remained stable, but its use for reviews of laboratory results, follow-up monitoring visits, and medication adherence reminders or check-ins increased substantially.^9^

Regarding resources required to increase and help address barriers to biomarker/genomic testing in the US, oncologists identified broader insurance coverage, reflex testing, improved education, and lower testing costs as most important. According to Levit et al,^6^ innovative solutions are needed to close gaps in cancer care in rural areas, urban clusters, and urbanized areas, such as using the hub-and-spoke Project Extension for Community Healthcare Outcomes model, decreasing disparities in clinical trials by enrolling a more diverse population, providing expansion of cancer care services and transportation to decrease patient travel time, addressing insurance restrictions, increasing access to clinical trials, and creating a cohesive partnership between providers and their communities to aid in cancer prevention and treatment. These suggestions reflect the fact that rural communities and patient populations are not homogenous. Other suggestions included ensuring that reimbursement is linked to adherence of biomarker testing guidelines, better coding for the reimbursement system, and reducing the Medicare 14-day rule, which slows testing rates. Processes introduced to encourage reflex testing, incorporation of recommendations into clinical decision support systems (to support, encourage, and incentivize best practices^7^), and better collaboration and communication between the oncology care team and pathologists to enable culture change and to educate and empower patients^10^ may also help address barriers to testing in rural areas and urban clusters.

### Limitations

The limitations of self-reported survey data include that they may not align with medical records or administrative health claims data, such as biomarker testing rates. Oncologists may also generalize their experiences across cancer types instead of focusing on mCRC-specific insights. Oncologists who responded spent most of their time in urban clusters. According to the American Society of Clinical Oncology, 66% of rural counties have no oncologist serving the area.^11^ In addition, only 11.6% of oncologists practice in these areas.^12^ This highlights the existing “oncology deserts” and the lack of ­oncologists who practice solely in rural settings who could participate in our survey. Nevertheless, as the mean time spent in rural areas was 18%, some respondents spent more than approximately one-fifth of their time in this setting. Some oncologists practice at locations with both urban and rural patients, in which case isolating responses specific to rural patients may be difficult. Survey results may not be generalizable to a wider population but highlight the unmet need for biomarker testing in rural settings.

## Conclusion

Overall, oncologists from rural areas and urban clusters reported being familiar with biomarker testing in mCRC. However, reported biomarker testing is suboptimal in these areas, falling short of current guideline recommendations.^4,5^ Further exploration of rural biomarker testing practices is needed to understand the disparities, raise awareness, and develop strategies to improve testing and optimize patient care despite socioeconomic and geographic differences.

## Supplementary Material

oyad244_suppl_Supplementary_Material

## Data Availability

The data underlying this article will be shared on reasonable request to the corresponding author.
