# Supplementary material for: Biomarker Testing Trends in Patients With Metastatic Colorectal Cancer Who Live in Rural Areas and Urban Clusters in the US
Source: Oncologist. 2023 Sep 8;28(11):e1118–22. doi: 10.1093/oncolo/oyad244 (PMC10628579; doi:10.1093/oncolo/oyad244)

# Supplemental Content

**Biomarker Testing Trends in Patients With Metastatic Colorectal Cancer Who Live in Rural Areas and Urban Clusters in the United States**

Mark A. Lewis, MD, Lindsay Stansfield, PharmD, John M. Kelton, PharmD, and
Christopher H. Lieu, MD

# MATERIALS AND METHODS

## Study Design and Data Source

A 33-item, 25-minute, double-blinded, quantitative online survey was administered to oncologists who met the inclusion criteria. IQVIA’s proprietary panel of oncologists and trusted partner panels were used to identify board-certified oncologists who spent ≥40% of their time providing direct care in urban clusters (geographic area of 2,500 to 49,999 people, as defined by the US Census Bureau)^17^ or rural areas (all population, housing, and territories not included within an urbanized area or an urban cluster) and who had treated ≥2 patients with stage IV mCRC in the month prior to the survey. After screening, a subset of those who completed the quantitative survey participated in the qualitative survey (a 30-minute, web-assisted telephone interview). Invited participants provided their signed consent to participate and were paid for their time completing the survey. The protocol was reviewed and approved by the Western Institutional Review Board.

Oncologists who spent a proportion of their time practicing in urbanized areas (geographic area of ≥50,000 people) were allowed due to the “oncology deserts” that exist. Oncologists in Maine, Vermont, and West Virginia were excluded due to state legislation; those practicing 100% of their time in urbanized areas and those employed by the US government, Veterans Affairs, or Kaiser Permanente were also excluded.

The quantitative survey was conducted from February 12 to March 18, 2021, inclusive. The qualitative survey was conducted from June 16 to 29, 2021, inclusive. The quantitative survey consisted of questions regarding patient caseload; education, training, and experience in biomarker and genomic testing; barriers to biomarker and genomic testing; use of telehealth; and practice and patient demographics. The qualitative survey interview questions targeted 6 areas: clinical practice description, biomarker and genomic testing patterns, pathology, and molecular tumor board, tumor tissue journey, electronic health records, and training/educational opportunities.

## Study Population

Study participants were medical oncologists or hematologists/medical oncologists who spend ≥40% of their time providing direct patient care in urban clusters or rural areas and who had treated ≥2 patients for mCRC in the prior month. Oncologists practicing 100% of their time in urbanized areas were excluded. The qualitative survey included a subset of the oncologists who had previously responded to the quantitative survey and had expressed interest in completing the qualitative survey.

## Measures of Statistical Interest

Descriptive statistics were used to characterize the data. All variables of interest were reported descriptively for all oncologists who responded, and these variables included n (%) for the categorical variables and the mean, median, standard deviation, minimum, and maximum number for continuous variables, collectively providing an interpretation of the distributional characteristics of the data. Qualitative survey interviews were analyzed manually, grouped by common themes, and reported descriptively with direct quotations from oncologists on key themes.

**SUPPLEMENTAL FIGURE 1.** Patient demographics


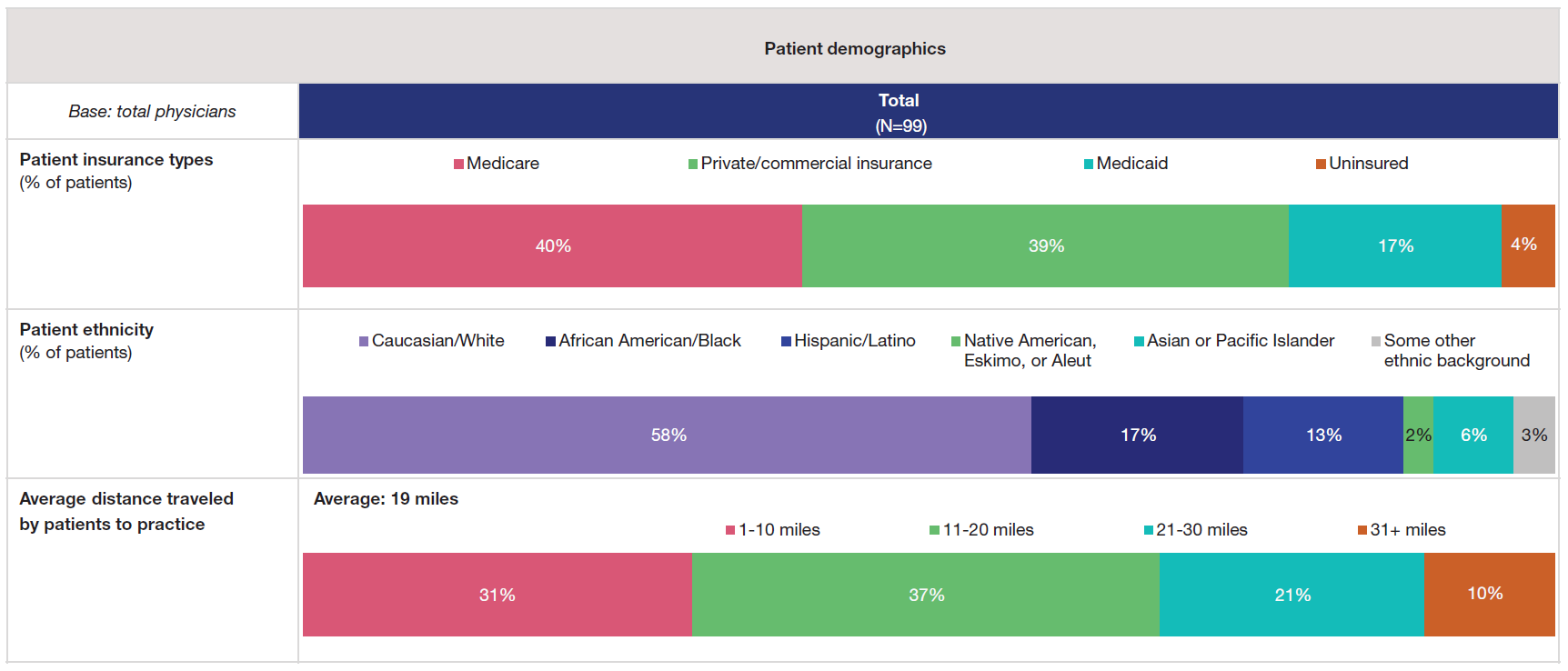

Supplement: oyad244_suppl_Supplementary_Material [file oyad244_suppl_supplementary_material.zip › Supplemental Content.docx]
